# Magnetic Luffa-Leaf-Derived Hierarchical Porous Biochar for Efficient Removal of Rhodamine B and Tetracycline Hydrochloride

**DOI:** 10.3390/ijms232415703

**Published:** 2022-12-11

**Authors:** Yingjie Su, Yangyang Zheng, Meiqin Feng, Siji Chen

**Affiliations:** 1 College of Life Sciences, Jilin Agricultural University, Changchun 130118, China; 2 Key Laboratory of Straw Comprehensive Utilization and Black Soil Conservation, Ministry of Education, Jilin Agricultural University, Changchun 130118, China

**Keywords:** lignocellulose, magnetic biochar, hierarchical porous, organic pollutants, efficient removal

## Abstract

Luffa leaf (LL) is an agricultural waste produced by loofah. In this work, LL was used as biomass carbon source for biochars for the first time. After carbonization, activation, and chemical co-precipitation treatments, a magnetic lignocellulose-derived hierarchical porous biochar was obtained. The specific surface area and total pore volume were 2565.4 m^2^/g and 1.4643 cm^3^/g, and the surface was rich in carbon and oxygen functional groups. The synthetic dye rhodamine B (RhB) and the antibiotic tetracycline hydrochloride (TH) were selected as organic pollutant models to explore the ability to remove organic pollutants, and the results showed good adsorption performances. The maximum adsorption capacities were 1701.7 mg/g for RhB and 1755.9 mg/g for TH, which were higher than most carbon-based adsorbents. After 10 cycles of use, the removal efficiencies were still maintained at more than 70%, showing good stability. This work not only verified the feasibility of lignocellulose LL as a carbon source to prepare biochar but also prepared a magnetic hierarchical porous adsorbent with good performances that can better treat RhB and TH, which provided a new idea and direction for the efficient removal of organic pollutants in water.

## 1. Introduction

With the rapid development of global industrialization, the problem of water pollution has attracted a lot of attention worldwide [1]. Various organic pollutants, such as organic dyes and antibiotics, have been detected in water bodies [2,3]. They are not only difficult to degrade naturally. They can cause teratogenesis, cancer, and death [4,5]. Traditional treatment methods, such as chemical precipitation [6], electrocatalysis [7], and microbial degradation [8], struggle to completely remove these impurities. Electrocatalysis, for example, can be costly due to using more expensive metals as electrodes [7]. The chemical precipitation method causes serious secondary pollution due to its use of a large number of flocculants [6]. Although the microbial method has low energy consumption and is sustainable, the treatment cycle is longer than for other methods [8]. Therefore, there is an urgent need to develop more efficient and green methods to quickly remove organic pollutants. The adsorption method has become a research hotspot in recent years due to its advantages of simple operation, mild conditions, and no secondary pollution [9,10]. The adsorbent, as a key index to measure the adsorption efficiency and evaluate the effect of an adsorption treatment, is one of the important parameters affecting the adsorption process.

In addition to some highly available sustainable biomass that is often used as adsorbents due to its excellent adsorption properties [9,10], carbon-based materials such as graphene [11], carbon nanotubes [12], and highly poly carbon fibers [13] also show great adsorption potential. However, such carbonaceous materials are not only extremely expensive but are also difficult to obtain. Biochar is a kind of carbon-rich product generated by the pyrolysis of biomass under anaerobic conditions [14]. Its advantages of easy access to carbon sources and low cost have given it a popular role in a new class of carbon-based materials [14]. Generally speaking, the properties of biochar are mainly determined by its natural structure and preparation process. The unique natural structure and more advanced preparation techniques often maximize the potential of biochar properties, which surprises researchers [15,16]. Lignocellulose is often used as one of the main carbon sources for biochar preparation and is composed of cellulose, hemicellulose, and lignin [17,18]. These lignocellulosic components intertwine with each other, forming complex structures [18] and eventually becoming organs with more attractive structures, such as flowers, rods, and leaves, through the process of biological differentiation during plant growth. Our team has been working on the development and utilization of lignocellulosic resources for nearly ten years [19,20,21], but until now, these valuable resources have not been thoroughly explored.

Towel gourd (*Luffa cylindrica*) is one of the most important melon vegetables. Due to its rich nutrition, high yield, and good economic benefits, loofah is not only widely planted throughout China but is also widely cultivated in the temperate and tropical regions of the world [22]. With the increasing demand for loofah and the continuous development of cultivation technology, the planting scale of loofah is expanding, and the agricultural waste produced by loofah is also increasing. Luffa leaf (LL) is a kind of agricultural waste produced by the cultivation of loofah. The main component is lignocellulose [23]. Although there have been some reports on LL in recent years, most of them have focused on the medicinal use of its extracts [24,25]. Few studies have reported on biochar preparation from LL, and its performance was not clear, which gives us great motivation to continue to exploit this valuable and abundant lignocellulosic resource.

In this work, LL was used as a carbon source to prepare hierarchical porous biochars. Three bases, including NaOH, KOH, and a mixed base (MB, consisted of equal masses of NaOH and KOH), were used to explore the best preparation activator and the influences of different activators on the preparation of biochars. In order to improve the stability and rapid recovery ability of LL-based hierarchical porous biochar (LLB), magnetic nanoparticles (Fe_3_O_4_) were loaded via a chemical co-precipitation method. Subsequently, the antibiotic tetracycline hydrochloride (TH) and the organic synthetic dye rhodamine B (RhB) were chosen as pollutant models to explore the removal efficiency of biochars in a water environment. Finally, the adsorption mechanism was also studied and discussed. The focus of this study was to explore the feasibility of using LL as a carbon source to prepare magnetic hierarchical porous biochar and to prepare a series of biochars with excellent performance, rapid recovery, and stable reproduction to provide effective ideas for the improved and more efficient removal of organic pollutants.

## 2. Results and Discussion

### 2.1. Preparation of Magnetic Biochar

**Figure 1:**
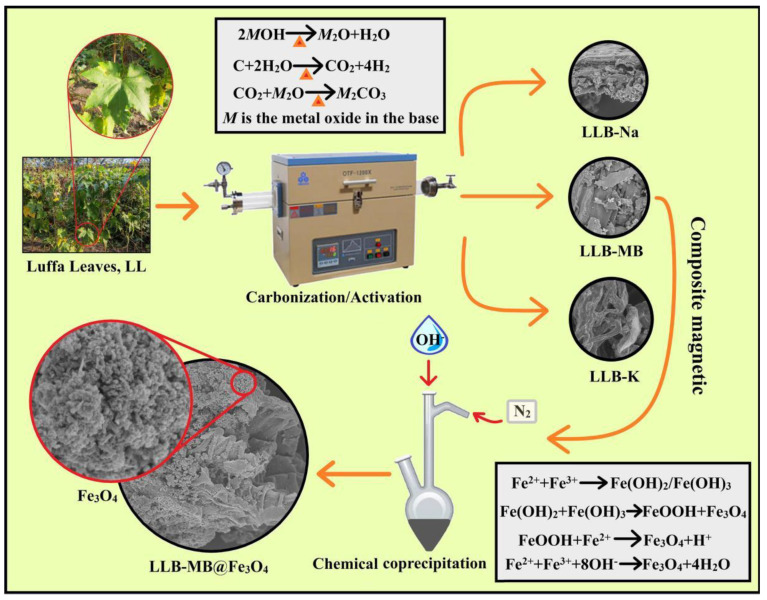
Scheme of the preparation of magnetic hierarchical porous biochar.

The preparation process of magnetic hierarchical porous biochar is shown in Figure 1 and can be divided into two steps: the preparation of hierarchical porous biochar and a magnetic composite. Of course, there were also two possible reaction mechanisms, including the MB activation process and chemical co-precipitation.

*MB Activation process* [26,27,28]: In the high-temperature pyrolysis process, MB generates M_2_O and water, and is then further ionized to form K^+^, Na^+^, and OH^−^. These ions migrate or insert carbon precursors and react with them to generate carbon dioxide and water. The carbon dioxide further reacts with the oxide produced by MOH (Na_2_O or K_2_O) to form carbonate (Na_2_CO_3_ or K_2_CO_3_), which etches the carbonized sample. Finally, microporous or mesoporous structures are formed in the carbon.

*Chemical co-precipitation* [29,30,31]: Under N_2_ protection, Fe^2+^ and Fe^3+^ form unstable colloidal compounds (Fe(OH)_2_ and Fe(OH)_3_) in the presence of OH^−^. These alternate compounds partially react with H^+^ to form magnetic nanoparticles and FeOOH. As the pH of the solution increases, excess OH^−^ continues to participate in the reaction, eventually forming Fe_3_O_4_.

### 2.2. Results of Characterizations

**Figure 2:**
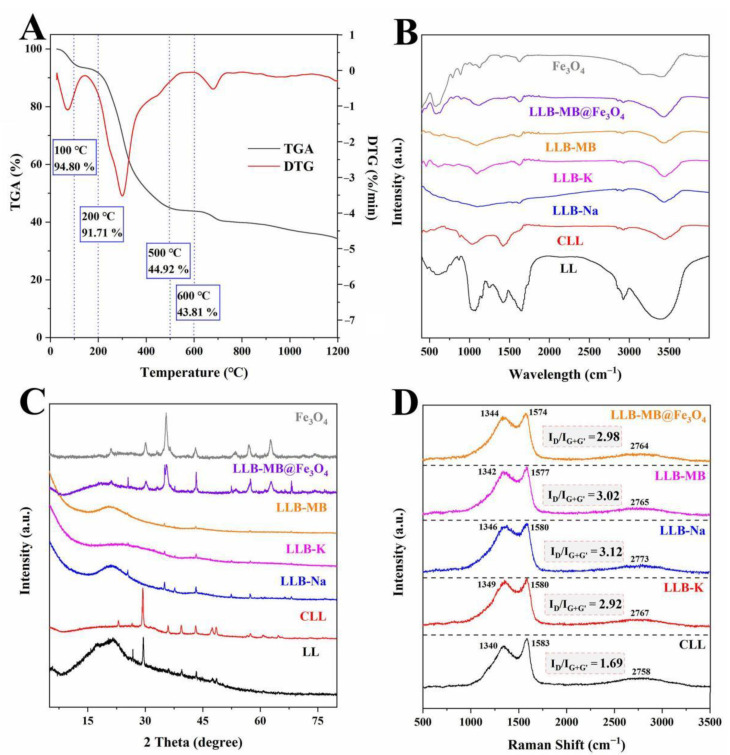
(**A**) TGA and DTG curves of LL. (**B**) FT-IR spectra, (**C**) XRD, and (**D**) Raman spectra of samples.

The results of the TGA and DTG curves, FT-IR spectra, XRD, and Raman spectra of the samples are shown in Figure 2. The effects of temperatures on LL were obtained by TGA and DTG tests under the protection of nitrogen with a heat rate of 10 °C/min, and the results are shown in Figure 2A. The TGA curves correspond to different stages of carbonized sample formation, indicating that there were three main stages of weight loss in the range from room temperature to 1200 °C. The first stage occurred from room temperature to 100 °C and was caused by the evaporation of water from the physical surface [32]. Then, the second stage occurred at 100–200 °C and may have been due to the loss of residual water from the pores inside LL [33]. The third stage started at 200 °C and ended at 500 °C. Like many types of lignocellulosic biomass [32,33,34], the main oxygenated components of LL were lignocellulose, which is composed of cellulose, hemicellulose, and lignin, and macromolecular organic materials such as proteins and fats [33,34]. These components can be decomposed into gas and tar during high-temperature pyrolysis and then removed, which causes significant weight loss. At 500 °C, the carbon yield was 44.92%. As the temperature continued to rise, the TGA and DTG curves did not show any further weight loss, especially when the temperature was 600 °C. The carbon yield was 43.81%, which was not much different from that at 500 °C. Therefore, considering the sustainable development and energy savings, the carbonization temperature of 500 °C was finally selected. 

The functional groups of the samples were analyzed by an FT-IR spectroscopy test, and the results are shown in Figure 2B. The wide band at 3250−3550 cm^−1^ represents the stretching vibration of the hydroxyl functional group (O-H) [32,33]. The bands at 2800−2900 cm^−1^ represent the symmetric and asymmetric stretching vibrations of the -CH, -CH_2_, and -CH_3_ groups [27,28,29]. The band at 1680−1720 cm^−1^ represents the stretching vibration of C=O [33]. The band at 1300−1400 cm^−1^ represents the C-H symmetric bending vibration of the methyl group. The band at 1200−1250 cm^−1^ represents the deformation vibration of the methylene group [32]. The bands around 980−1150 cm^−1^ represent the stretching vibration of the C-O in alcohols, phenols, acids, or esters [32,33]. The characteristic bands of Fe-O groups on Fe_3_O_4_ were observed at 575−585 cm^−1^ [26]. Thus, it can be inferred that Fe_3_O_4_ particles were successfully loaded on LLB-MB@Fe_3_O_4_.

The crystal structure of the samples was analyzed by XRD, and the results are shown in Figure 2C. The wide peak around 23° of LL was typical of lignocellulose [19,20]. Other irregular and obvious bands may be related to inorganic salts. The irregular and obvious bands of CLL were more prominent after carbonization, which might be due to salting out during the high-temperature pyrolysis process. After activation (regardless of the activators) these bands became weaker or even partially disappeared, largely due to the washing process during the preparation. In addition, from LLB-MB@Fe_3_O_4_ samples, characteristic peaks representing Fe_3_O_4_ can be clearly seen, such as (111), (220), (311), (400), (422), (511), and (440) [35,36], indicating successful magnetic recombination.

The presence of defects in the biochars was tested by Raman spectra, and the results are shown in Figure 2D. Three typical peaks obtained from the results, including the D-band with amorphous carbon around 1340−1349 cm^−1^ [37], the G-band with graphitic carbon around 1574−1583 cm^−1^ [38], and the G′-band (also named the 2D-band), which was sensitive to the number of graphene layers, had no defect and no D-peak, and could be fitted to a single peak of a single layer of graphene or to multiple peaks of a multilayer of graphene, which was caused by the double resonant Raman scattering with two-phonon emissions around 2758−2773 cm^−1^ [37,38]. To measure the degree of defect and disorder in carbons, the intensity ratio of the D-band and G-band with the G′-band (I_D_/I_G+G′_) was used as an important index. The I_D_/I_G+G′_ value of CLL was 1.69. After activation, the I_D_/I_G+G′_ values of LLB-Na and LLB-K were 2.92 and 3.12, which indicated that more amorphous carbon structures were generated in the biochars during activation when using NaOH or KOH as activators. Coincidentally, the I_D_/I_G+G′_ value of LLB-MB was 3.02, which was lower than LLB-Na and higher than LLB-K. After magnetic recombination, the I_D_/I_G+G′_ value of LLB-MB@Fe_3_O_4_ was 2.98.

**Figure 3:**
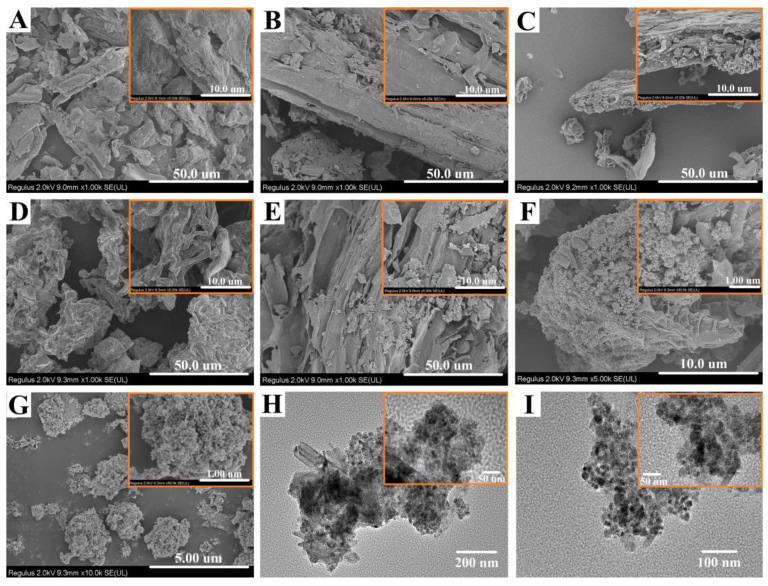
(**A**) SEM images of (**A**) LL, (**B**) CLL, (**C**) LLB-Na, (**D**) LLB-K, (**E**) LLB-MB, (**F**) LLB-MB@Fe_3_O_4_, and (**G**) Fe_3_O_4_. TEM images of (**H**) LLB-MB@Fe_3_O_4_ and (**I**) Fe_3_O_4_.

The morphology of the samples was observed by SEM and TEM, and the results are shown in Figure 3. LL showed obvious lignofibers with a large number of lamellar structures. It was also clear that there were significant macropores, which may be solvent channels in the process of plant growth [19,20]. After carbonization, the surface of CLL became rough and compact due to intense dehydration, and there were obvious folds and furrows. Figure 3C–E show LLBs treated with different activators, which had visible differences, such as the degree of fragmentation and etching. This result indicated that different activators, including MB, had different effects on biochar preparation processes. After recombination, a large number of regular particles appeared on the surface of LLB-MB@Fe_3_O_4_ (Figure 2F), indicating that the chemical co-precipitate method was indeed successful in loading Fe_3_O_4_ onto the biochar. To further confirm the successful recombination, Fe_3_O_4_ (Figure 3I) and LLB-MB@Fe_3_O_4_ (Figure 3H) were tested by TEM, supporting this conclusion.

**Figure 4:**
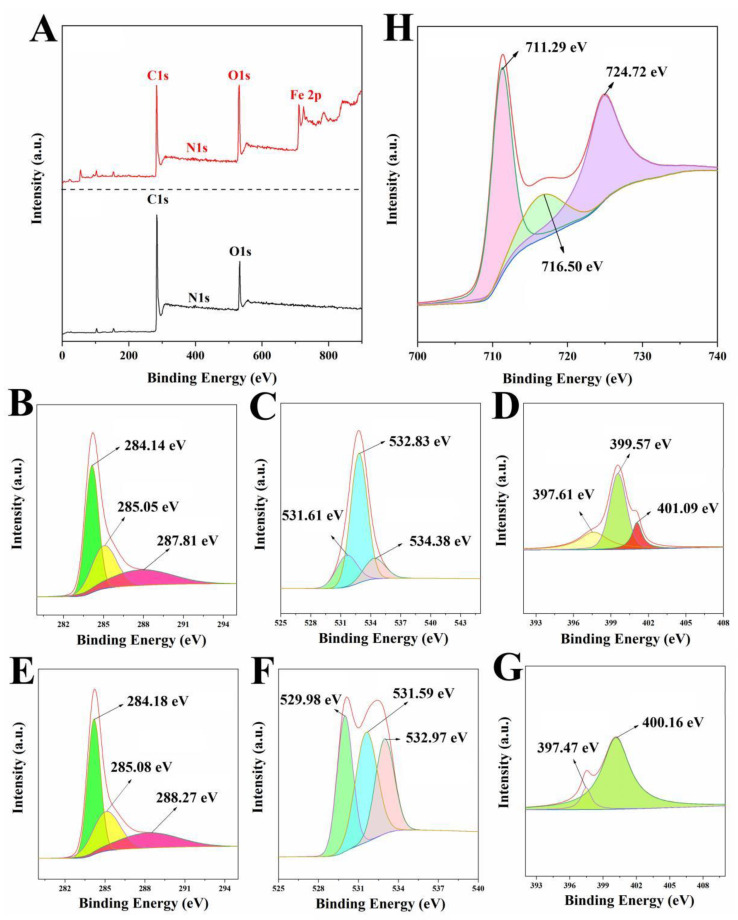
(**A**) XPS spectra of LLB-MB and LLB-MB@Fe_3_O_4_. The C1s, O1s, and N1s of LLB (**B**−**D**) and LLB-MB@Fe_3_O_4_ (**E**−**G**). (**H**) The Fe 2p of LLB-MB@Fe_3_O_4_.

The surface chemical and electronic states of the samples were tested by XPS spectroscopy, and the results are shown in Figure 4. LLB-MB contained mainly C (84.24%), O (13.89%), and N (1.87%) elements. Meanwhile, LLB-MB@Fe_3_O_4_ contained C (62.98%), O (26.5%), N (2.59%), and Fe (7.93%) elements. Further fitting these elements can obtain different functional keys. The high-resolution C1s spectrum of LLB-MB and LLB-MB@Fe_3_O_4_ showed three classical peaks, which were obtained at 284.14–284.18 eV, 285.05–285.08 eV, and 287.81–288.27 eV, corresponding to C-C, C-O, and C=O, respectively [32,33]. The high-resolution O1s spectrum of LLB-MB and LLB-MB@Fe_3_O_4_ had peaks at 529.98 eV, 531.59–531.61 eV, 532.83–532.97, and 534.38 eV, corresponding to quinones, C=O, C-O, and -OH, respectively [32,33]. The high-resolution N1s spectrum of LLB-MB and LLB-MB@Fe_3_O_4_ both had peaks at 397.41–397.61 eV and 399.57–400.16 eV, corresponding to pyridinic-N and pyrrolic-N [39]. Additionally, LLB-MB had a special peak at 401.09 eV corresponding to graphite-N [39], which may have disappeared during the process of the chemical co-precipitation of the composite magnetic properties.

**Figure 5:**
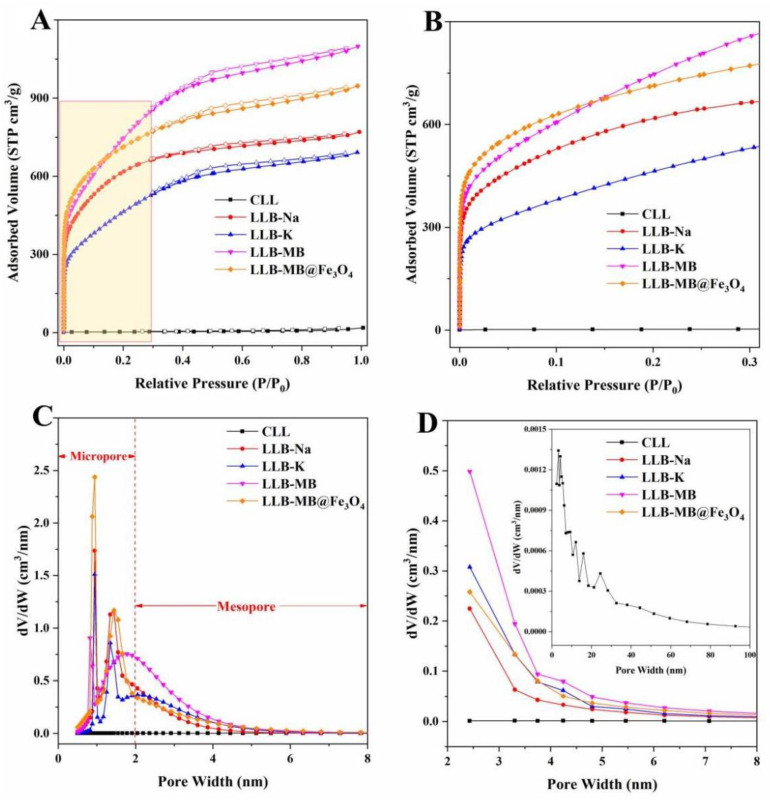
N_2_ adsorption–desorption isotherms of samples under relative pressures of (**A**) 0.0−1.0 and (**B**) 0.0−0.3. Pore distributions of samples based on the (**C**) NLDFT method and (**D**) BJH method.

A N_2_ adsorption–desorption isotherm was used to test the specific surface area and porosity of the samples, and the results are shown in Figure 5 and Table 1. The specific surface area and total pore volume of CLL were 9.0 m^2^/g and 0.0253 cm^3^/g, respectively, which were far from sufficient to support CLL as a porous biochar. Therefore, further activation treatment was needed to increase the porosity and improve the adsorption performance. The effect of alkali activation was not in doubt [27,28,29], so different activators, including NaOH, KOH, and MB, were used for the further processing of CLL. After activation, the results showed that all LLBs exhibited typical type IV isotherms with typical H3 hysteresis loops (Figure 5A) [40,41], which indicated the existence of mesopore structures in the prepared biochars. Additionally, all LLBs had significant capillary absorption at the relative pressure range of 0.0 to 0.3 (Figure 5B), which was strong proof of the existence of a microporous structure [19]. Meanwhile, the specific surface areas and total pore volumes of LLB-Na, LLB-K, and LLB-MB were 2139.2 m^2^/g and 1.1886 cm^3^/g, 1500.8 m^2^/g and 1.0692 cm^3^/g, and 2446.6 m^2^/g and 1.6990 cm^3^/g, respectively. It can be seen from the above data that different activators indeed had different activation behaviors, which caused different activation results. Beyond that, the specific surface area and total pore volume of LLB-MB were obviously greater than those of LLB-Na and LLB-K, which indicated that MB-mediated activation was more effective than single-base activation for CLL. More notably, after magnetic recombination, the surface area of LLB-MB@Fe_3_O_4_ (2565.4 m^2^/g) was again increased compared with LLB-MB, which can be explained by the presence of uniformly loaded magnetic nanoparticles that improved the specific surface area of biochar [28]. On the other hand, the successful loading of Fe_3_O_4_ occupied part of the volume of the mesopore. Although the total pore volume decreased slightly (1.4643 m^3^/g), the proportion of capillary micropores increased, thus increasing the specific surface area of LLB-MB@Fe_3_O_4_, which can also be seen in Figure 5B. In addition, the pore size distribution results also showed the presence of both microporous and mesoporous structures, and combined with the macropores naturally presented in LL, it was concluded that LLB was successfully produced after activation (i.e., lignocellulose-derived hierarchical porous biochar). The mean pore sizes of LLB-Na, LLB-K, and LLB-MB were 2.22, 2.85, and 2.78 nm, which were larger than the molecular sizes of RhB (1.2 nm) and TH (1.3 nm), which can be used as adsorbents for efficient organic pollutant removal. At the same time, the mean pore size of LLB-MB@Fe_3_O_4_ was 2.28 nm, which was also applicable to the above conclusion.

### 2.3. Results of Adsorption Experiments

#### 2.3.1. Adsorption Kinetics

**Figure 6:**
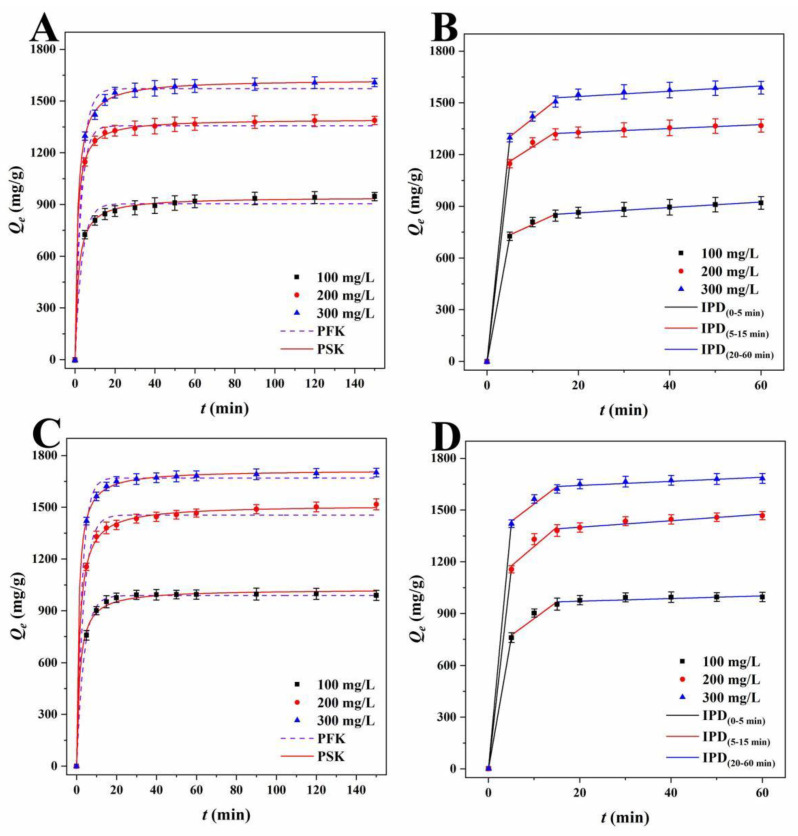
PFK and PSK plots of RhB for (**A**) LLB-MB and (**C**) LLB-MB@Fe_3_O_4_ at 303 K. IPD plots of RhB for (**B**) LLB-MB and (**D**) LLB-MB@Fe_3_O_4_ at 303 K.

**Figure 7:**
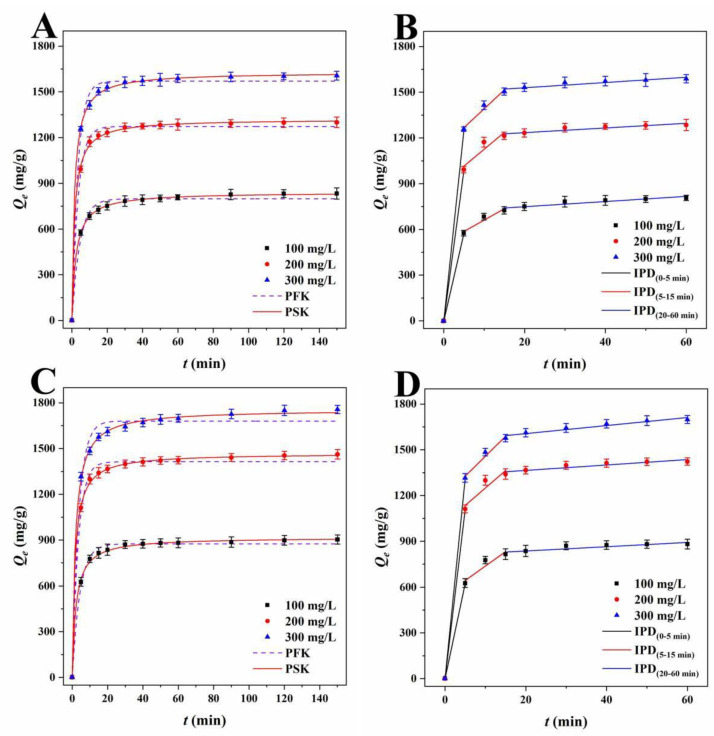
PFK and PSK plots of TH for (**A**) LLB-MB and (**C**) LLB-MB@Fe_3_O_4_ at 303 K. IPD plots of TH for (**B**) LLB-MB and (**D**) LLB-MB@Fe_3_O_4_ at 303 K.

Adsorption kinetics describe the changes in the adsorbent and model adsorbent with contact time [42]. The influence of contact time on the adsorption capacity of RhB and TH by LLB-MB and LLB-Mb@Fe_3_O_4_ was explored at different initial solution concentrations at 303 K, and the results are shown in Figure 6 and Figure 7. It can be seen that if the adsorbent was LLB-MB or LLB-Mb@Fe_3_O_4_ and if the pollutant was RhB or TH the overall trends of each adsorption process were similar. that is, the adsorption capacities rapidly increased in the first 15 min and then reached equilibrium in 60 min. Further increasing the adsorption time did not lead to an obvious increase in the adsorption amount. To study the control mechanism of the chemical reactions in the adsorption processes, three common adsorption kinetics models, including pseudo-first-order kinetic (PFK, Equation (1)), pseudo-second-order kinetic (PSK, Equation (2)), and intraparticle diffusion models (IPD, Equation (3)), were used to analyze the adsorption kinetic data (Table 2 and Table 3), shown as follows:

(1) $$\ln(Q_{e}-Q_{t})=\ln Q_{e}-k_{1}t$$

(2) $$\frac{t}{Q_{t}}=\frac{1}{k_{2}Q_{t}^{2}}+\frac{t}{Q_{e}}$$

(3) $$Q_{t}=k_{3}t^{0.5}+C$$

where *Q_t_* (mg/g), *C* (mg/g), *k*_1_ (min^−1^), *k*_2_ (g mg^−1^ min^−1^), and *k*_3_ (mg g^−1^ min^−0^.^5^) represent the adsorption capacities of samples at different time points (*t)*; the thickness of the boundary layer; and the PFK, PSK, and IPD adsorption kinetic rate constants, respectively.

Lagergren’s PFK model was used to investigate the adsorption process, and the correlation coefficients (*R*^2^) of LLB-MB and LLB-MB@Fe_3_O_4_ ranged from 0.9842 to 0.9957 and from 0.9451 to 0.9987 for RhB. The PFK correlation coefficients (*R*^2^) of TH were 0.9450−0.9953 and 0.9863−0.9939 for LLB-MB and LLB-MB@Fe_3_O_4_. Although the theoretical adsorption capacities (*Q_e.cat_)* calculated from the model (904.1, 1356.3, and 1571.5 mg/g of LLB-MB for RhB; 988.3, 1415.9, and 1670.0 mg/g of LLB-MB@Fe_3_O_4_ for RhB; 798.3, 1237.1, and 1570.1 mg/g of LLB-MB for TH; and 874.8, 1413.7, and 1680.4 mg/g of LLB-MB@Fe_3_O_4_ for TH) were slightly lower than the experimental *Q_e_* (941.6, 1386.6, and 1606.5 mg/g of LLB-MB for RhB; 998.1, 1502.2, and 1698.2 mg/g of LLB-MB@Fe_3_O_4_ for RhB; 831.4, 1298.0, and 1602.4 mg/g of LLB-MB for TH; and 896.1, 1453.5, and 1749.9 mg/g of LLB-MB@Fe_3_O_4_ for TH), they still indicated that the PFK model may be applicable to the adsorption process in some aspects. At the same time, it also showed that the adsorption capacity will increase with the initial concentration increasing to some extent [42,43]. While Ho-McKay’s PSK model was used to fit the data, the correlation coefficients (*R*^2^) of LLB-MB and LLB-MB@Fe_3_O_4_ ranged from 0.9987 to 0.9998 and from 0.9964 to 0.9998 for RhB and ranged from 0.9989 to 0.9998 and from 0.9983 to 0.9994 for TH, which showed the applicability of PSK in the adsorption process. In addition, the adsorption processes of LLB-MB and LLB-MB@Fe_3_O_4_ for RhB and TH may be chemical reactions, and the adsorption behaviors of chemisorption bonds formed by transfer, exchange, or sharing can control the adsorption rate and affect the adsorption process of the adsorbents [28,42,43].

As for the Weber–Morris IPD model, which was used to fit the experimental data (Table 3), if the curve of *Q_t_* relative to *t^0.5^* is a straight line through the origin, then it can be speculated that IPD should be an important step in determining the adsorption rate [27]. However, no single linearity can be observed in Figure 6B,D or Figure 7B,D but rather three linear regions with different adsorption rate constants. In the first stage (0−5 min), the rapid adsorption of LLB-MB and LLB-MB@Fe_3_O_4_ was due to the abundant adsorption sites on the outer surface. At this stage, the mass transfer behavior of the boundary layer diffusion was considered possible [27]. The second stage (5−15 min) was the IPD stage. At this stage, RhB or TH gradually entered the pore and bound to the inner surface of LLB-MB and LLB-MB@Fe_3_O_4_. The diffusion resistance caused by the small pore size of the adsorbent reduced the adsorption rate. In the third stage (20−60 min), the diffusion resistance continued to increase, and the adsorption rate continued to decrease and finally reached the adsorption equilibrium. The above results indicated that IPD played an important role in the adsorption process of RhB and TH. However, in the second and third adsorption stages, the linear fitting curves did not cross the origin, so it was not the only factor affecting the adsorption processes [27]. 

#### 2.3.2. Adsorption Isotherm

**Figure 8:**
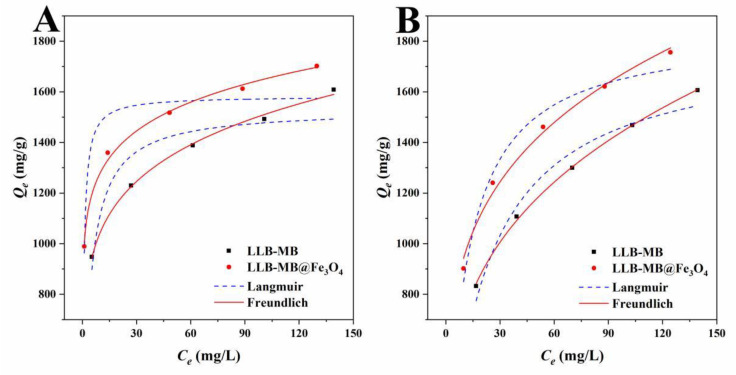
Langmuir and Freundlich isotherms of (**A**) RhB and (**B**) TH for LLB-MB and LLB-MB@Fe_3_O_4_ at 303 K.

The effect of concentration on adsorption capacity of adsorbent has usually been studied by studying adsorption isotherms [44,45]. The adsorption behaviors of adsorbents (LLB-MB and LLB-MB@Fe_3_O_4_) and adsorbates (RhB and TH) at 303 K were investigated with different initial solution concentrations, and the results are shown in Figure 8. It can be seen from the results that the adsorption capacities of the adsorbents increased with increases in the initial concentrations of the solutions. The adsorption isotherm data (Table 4) were investigated using the Langmuir isotherm model (Equation (4)) and the Freundlich isotherm model (Equation (5)):

(4) $$\frac{C_{e}}{Q_{e}}=\frac{C_{e}}{Q_{m}}+\frac{1}{Q_{m}K_{L}}$$

(5) $$lnQ_{m}=\frac{1}{n}\ln C_{e}+\ln K_{F}$$

where *Q_m_* (mg/g), *K_L_* (L/mg), and *K_F_* (mg g^−1^(L mg^−1^)^1/n^) represent the maximum adsorption capacity of sample, calculated by the adsorption isotherm model, the Langmuir model, and the Freundlich adsorption isotherm constants, respectively.

The Langmuir isotherm model was often used to describe the adsorption of homogeneous monolayer molecules, while the Freundlich isotherm model was used to analyze heterogeneous multilayer adsorption processes. The Langmuir correlation coefficients (*R*^2^) were 0.8738 for RhB and 0.9662 for TH for LLB-MB and were 0.8637 for RhB and 0.9728 for TH for LLB-MB@Fe_3_O_4_. The Freundlich correlation coefficients (*R*^2^) were 0.9972 and 0.9991 for LLB-MB for RhB and TH, and 0.9957 and 0.9912 for LLB-MB@Fe_3_O_4_ for RhB and TH. Meanwhile, the intensity factors (*n_F_)* of LLB-MBand LLB-MB@Fe_3_O_4_ were 6.30 and 9.13 for RhB, and 3.27 and 4.01 for TH, respectively. In conclusion, it can be inferred that the adsorption processes of RhB and TH were not simple single-layer adsorption processes but were likely to accumulate in heterogeneous multilayer adsorption [44,45,46].

#### 2.3.3. Adsorption Thermodynamics

**Figure 9:**
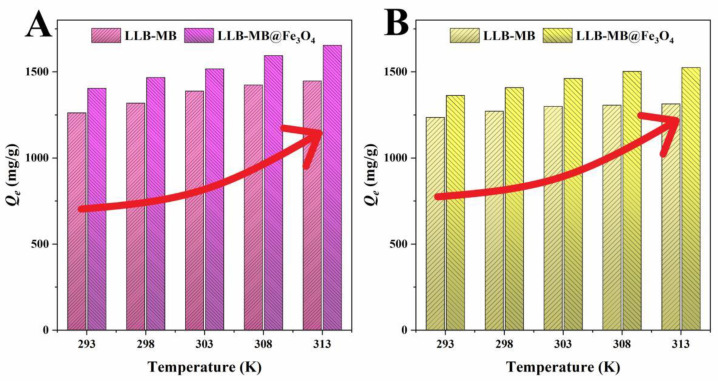
Adsorption thermodynamics of (**A**) RhB and (**B**) TH for LLB-MB and LLB-MB@Fe_3_O_4_.

Temperature was one of the most important factors affecting adsorption, which usually affected the adsorption process by influencing the thermal motion of molecules and promoting or inhibiting the formation of chemical bonds [47]. The effects of different temperatures (293, 298, 303, 308 K, and 313 K) on the adsorption of organic pollutants by LLB-MB and LLB-MB@Fe_3_O_4_ are shown in Figure 9. With the increase in temperature from 293 K to 313 K, the adsorption capacities were both increased. Obviously, the temperature increase promoted the adsorption process. That is, the influence of a high-temperature environment on the adsorption of organic pollutants (RhB and TH) by biochar adsorbents (LLB-MB and LLB-MB@Fe_3_O_4_) was favorable. 

To further describe the effects of temperatures on the adsorption process, the data were analyzed by the thermodynamic parameters (Table 5). The thermodynamic equations are as follows:

(6) $$ln\left(K_{T}\right)=-\frac{\Delta H}{RT}+\frac{\Delta S}{R}$$

(7) $$K_{T}=\frac{q_{e}}{C_{e}}$$

(8) ΔG=ΔH−TΔS

where Δ*S* (J mol^−1^ K^−1^) and Δ*G* (kJ/mol) represent the thermodynamic parameters standard entropy and standard free Gibbs energy, while Δ*H* (kJ/mol) and *R* represent the standard enthalpy and the gas constant (8.314 J/K·mol), respectively. 

It was not difficult to find that if the adsorbent was LLB-MB or LLB-MB@Fe_3_O_4_ and if the model pollutant was RhB or TH, the adsorption process was spontaneous, because all the Δ*G* values were negative [48]. The thermodynamic enthalpy (Δ*H)* values for the LLB-MB and LLB-MB@Fe_3_O_4_ adsorption were 16.23 and 26.98 kJ/mol for RhB and were 6.47 and 15.45 kJ/mol for TH, which further confirmed the endothermic property of the adsorption process. At the same time, the absolute values of the enthalpy change of all adsorbents were greater than 4.2 kJ/mol, in the range of 6.78−10.07 kJ/mol, which further indicated the existence of chemisorption in the adsorption process [49]. The Δ*S* values of adsorption were all positive, which indicated that the randomness and chaos degree of the interface between hierarchical porous biochars and solutions increased with the increasing temperatures [32,33,50].

#### 2.3.4. Effect of pH

**Figure 10:**
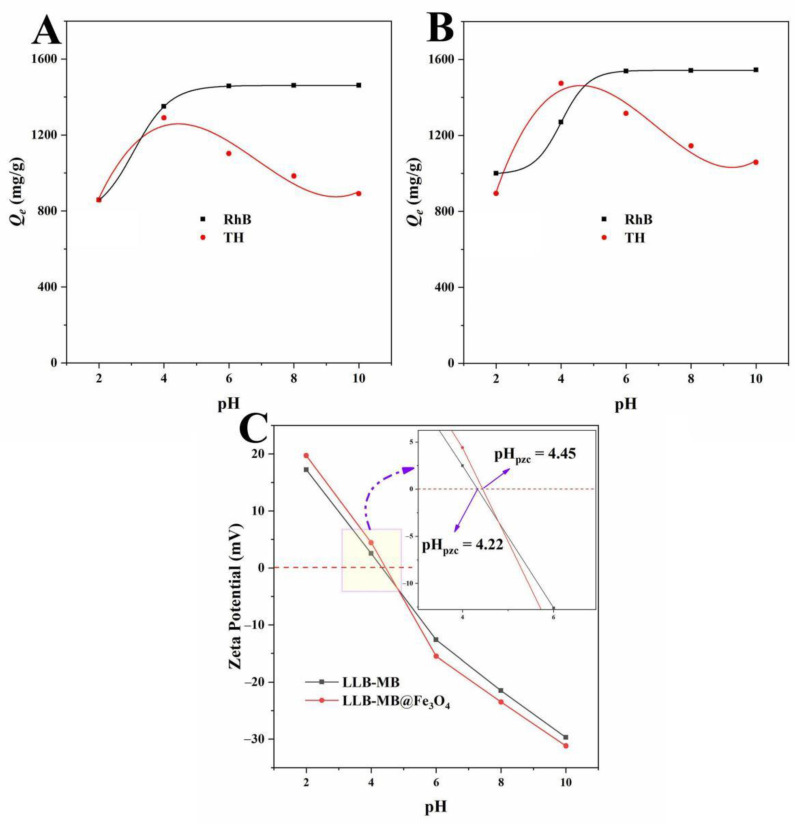
Effects of pH on the adsorption capacities of (**A**) LLB and (**B**) LLB-MB@Fe_3_O_4_. (**C**) Zeta potentials of LLB and LLB-MB@Fe_3_O_4_.

The pH value of the solution environment was an important factor affecting the adsorption process by affecting the surface properties of the adsorbents and the chemical properties of the adsorbed pollutant molecules to promote or inhibit the adsorption capacities [32,33]. The effect of pH on the adsorbents’ (LLB-MB and LLB-MB@Fe_3_O_4_) abilities to remove organic pollutants was studied in the pH range from 2 to 10, and the results are shown in Figure 10. With increases in the initial pH values, the adsorption capacities of LLB-MB and LLB-MB@Fe_3_O_4_ for RhB increased and then stabilized, while the TH removal capacities increased and then decreased. This phenomenon can be explained by the fact that RhB is a cationic dye, and the presence of a large amount of H^+^ in the solution at a lower pH value will compete with RhB for adsorption sites on the surfaces of biochars [27,28]. In addition, when the pH value of a solution is less than the pH_pzc_ of biochars (LLB-MB = 4.22 and LLB-MB@Fe_3_O_4_ = 4.45), the surfaces of biochars were positively charged and had electrostatic repulsion to RhB. With increases in pH, especially while the pH was greater than pH_pzc_, the surfaces of biochars started to become negatively charged, and the electrostatic attraction between the biochar and the cationic dye promoted the adsorption process. However, the enhancement of the RhB adsorption capacity by higher pH was not endless. When the pH value was greater than 6, increasing the pH value did not continue to significantly improve the RhB removal abilities of biochars, which indicated that electrostatic attraction was not the only force affecting the adsorption of cationic dye by biochars. For the adsorption of TH by biochars, pH had a more obvious influence, probably because it not only affected the surface electrochemical performance of biochars but also had great influences on the adsorbed molecular properties of TH. That is, TH existed in different forms (TH^+^, TH^0^, TH^−^, and TH^2−^) under different pH values [34,51]. When the pH value was lower than 3.30, TH^+^ was dominant and the zeta potential of biochars was positive. At this time, the mutually repulsive electrostatic attraction inhibited the adsorption processes. When the pH was higher than 3.30 and lower than 7.68, TH existed in the form of TH^0^ or TH^+−^. When the pH was greater than 7.68 and less than 9.68, TH existed in the form of TH^−^. Further, when the pH was greater than 9.68, TH was dominated by TH^2−^, which was not conducive to the adsorption of biochars with negative charges.

#### 2.3.5. Results of Cycle Tests

**Figure 11:**
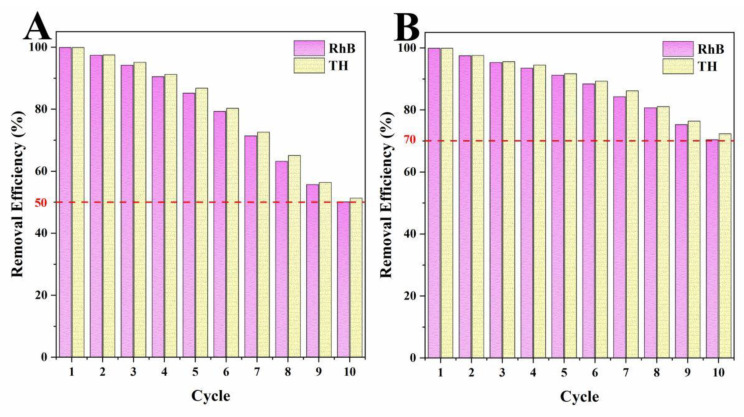
Cycle tests of (**A**) LLB-MB and (**B**) LLB-MB@Fe_3_O_4_.

The regeneration capacities of adsorbents were one of the most important parameters to evaluate the practical performance of biochar adsorbents [34,51]. Through the cycle tests, the cycling abilities of biochars to remove organic pollutants over ten cycles was tested, and the results are shown in Figure 11. By increasing of the number of cycles, the removal abilities of LLB-MB and LLB-MB@Fe_3_O_4_ decreased significantly. Although their removal efficiencies were significantly reduced, this reduction is inevitable for most lignocellulosic biochar. This may be caused by two reasons. On one hand, with the increase in cycles, the organic pollutants adsorbed by biochars form by-products on their surfaces in the process of re-carbonization, which may lead to a reduction in adsorption sites [28,51]. On the other hand, with increasing re-carbonization times the porous structures and surfaces of biochars become more fragile, which further affects the adsorption performance [39]. After 10 cycles, the RhB and TH removal efficiencies of LLB-MB were 50.1% and 51.3%, and the RhB and TH removal efficiencies of LLB-MB@Fe_3_O_4_ were 70.4% and 72.3%. Compared with LLB-MB, the cycle stability of LLB-MB@Fe_3_O_4_ was significantly higher. It can be speculated that the presence of magnetic nanoparticles may give the structure of LLB-MB@Fe_3_O_4_ higher mechanical strength than LLB-MB [39], either during adsorption or during re-carbonization. 

### 2.4. Probable Adsorption Mechanisms

**Figure 12:**
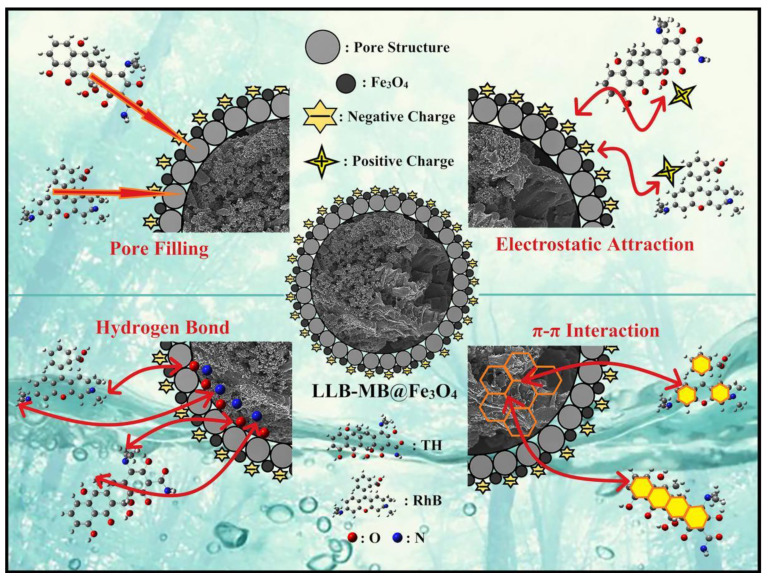
Probable adsorption mechanisms of LLB-MB@Fe_3_O_4_ for organic pollutant removal.

In this study, the large specific surface area and high total pore volume (2565.4 m^2^/g and 1.4643 cm^3^/g) of LLB-MB@Fe_3_O_4_ provided a large number of adsorption sites. Therefore, pore filling might exist and may have played an important role during the adsorption processes of efficient organic pollutant removal. In addition, there was a large number of unsaturated functional groups containing carbon, oxygen, and nitrogen on the surface of LLB-MB@Fe_3_O_4_, according to the results of the FT-IR and XPS tests. These functional bonds were likely to form hydrogen bonds between LLB-MB@Fe_3_O_4_ and RhB and TH molecules, which also could improve the removal ability. In addition, aromatic rings exist in LLB-MB@Fe_3_O_4_, which may also have π–π interactions with aromatic rings in RhB and TH to enhance the adsorption capacity. Finally, in the appropriate pH environment, there was a strong electrostatic attraction between LLB-MB@Fe_3_O_4_ and RhB and TH, which further promoted the adsorption process. According to some reported studies, magnetic nanoparticles may also exchange electrons with pollutant molecules to promote the chemisorption process. However, based on the above data in this work, the existence of this force was not confirmed, and we will focus on it in subsequent research. Thus, it can be speculated that LLB-MB@Fe_3_O_4_ exhibited a powerful ability for efficient organic pollutant (RhB and TH) removal in water under the joint action of pore filling, π–π interactions, H-bond interactions, and electrostatic attraction (Figure 12).

### 2.5. Comparison

The adsorption capacity was one of the most important parameters to evaluate the actual performance of the adsorbents. Therefore, the adsorption capacities of biochars were compared with those of other carbon-based adsorbents, including our previous reported results, and the results are shown in Table 6. It can be seen that LLB-MB and LLB-MB@Fe_3_O_4_ had some of the highest adsorption capacities among the carbon-based adsorbents, which fully indicated that the prepared biochars have great potential in wastewater treatment and can be used for efficient organic pollutant removal.

## 3. Materials and Methods

### 3.1. Materials and Reagents

In 2022, *Luffa* leaf (LL) was obtained from the experimental area of Jilin Agricultural University (Changchun, China), washed, dried, and crushed. NaOH, HCl, KOH, FeSO_4_, and FeCl_3_ were purchased from Beijing Chemical Works (Beijing, China) and used without further purification. RhB (CAS: 81-88-9, ≥95%) and TH (CAS: 64-75-5, 96%) were supplied by Aladdin Chemical (Shanghai) Co., Ltd. (Shanghai, China), and the structural formulas are shown in Figure S1 (Supplementary Material).

### 3.2. Preparation of LLBs

The obtained LL was carbonized at 500 °C for 60 min with a heating rate of 10 °C/min under the protection of nitrogen atmosphere. After the carbonized LL (CLL) cooled to room temperature, the CLL was mixed with a NaOH, KOH, or MB activator at a ratio of 4:1 and heated at 700 °C for 60 min. Then, the activated mixture was washed with HCl and deionized water until reaching a neutral pH. Afterwards, the biochars were dried and kept in a desiccator. The abbreviations LLB-K, LLB-Na, and LLB-MB represent the LL-based biochars activated by using KOH, NaOH, and MB as activators, respectively. 

### 3.3. Preparation of Magnetic Biochar

First, 1.0 g of biochar was added to a flask containing 50 mL of Fe^2+^ (5 mM) and Fe^3+^ (10 mM) under N_2_ protection. The reaction system was placed in an 80 °C ultrasonic oscillator. After mixing evenly, a 0.1 M NaOH solution was added to adjust the solution’s pH to 10.0 and hold for 30 min. After the mixture cooled to room temperature, the composite sample was recovered with a magnet, washed with deionized water to neutral, and dried in vacuum to a constant weight.

### 3.4. Adsorption Performances

In a batch adsorption experiment, 0.10 g/L of adsorbents were added to a flask containing organic pollutant solutions (RhB or TH). The flask was placed in a constant-temperature shaker at 150.0 RPM in the dark. After the adsorption process reached equilibrium, the suspension was centrifuged and diluted with the supernatant. The concentration of the solution was determined with an Agilent Cary-300 UV-vis spectrophotometer. The adsorption capacities of the samples were calculated with Equation (9):

(9) $$Q_{e}=\frac{(C_{0}-C_{e})\times V}{M}$$

where *Q_e_* (mg/g), *C_e_* (mg/L), *C*_0_ (mg/L), *M* (g), and *V* (L) represent the adsorption capacity of the adsorbent, the equilibrium concentration of the solution, the initial concentration of the solution, the mass of the adsorbent, and the volume of the solution, respectively.

The organic pollutant solutions were prepared at different concentrations (100, 200, and 300 mg/L), and 0.1 g/L of adsorbents were dispersed into flasks containing RhB or TH solutions at 303 K. Then, the concentrations of the solutions were determined at preset time intervals.

The organic pollutant solutions at different initial concentrations (100, 150, 200, 250, and 300 mg/L) were prepared and used to test the adsorption isotherm at 303 K. After adsorption saturation, the absorbances of the solutions were measured. 

The effects of temperatures (293, 298, 303, 308, and 313 K) on the adsorption capacity of the RhB and TH were investigated with the initial concentration of 200 mg/L with 0.1 g/L of adsorbents.

The variation in the adsorption capacities of the adsorbents with pH (2, 4, 6, 8, and 10) was also investigated. The solutions were adjusted with 0.1 M HCl and NaOH solutions.

### 3.5. Cycling Stability Studies

In each cycle, 1.0 g/L of adsorbents were placed into a flask containing 100 mg/L of organic pollutants. After adsorption, the recycled adsorbents were washed and re-carbonized at 500 °C under the protection of nitrogen atmosphere for 60 min. Then, the re-carbonized adsorbents were re-used as new adsorbents in the next cycle test.

### 3.6. Characterization Methods

Scanning electron microscopy (SEM, Hitachi S4800, Hitachi, Japan) and transmission electron microscope (TEM, FEI Tecnai G2 S-Twin F20, Hillsboro, USA) were used to examine the morphology of the materials. A thermogravimetric analysis of the samples was carried out under the protection of nitrogen flow (TGA, Netzsch STA409PC, Selb, Germany). An FT-IR spectrometer was used to characterize the surface functional groups of the materials between 400 and 4000 cm^−1^ at a resolution of 1 cm^−1^ (FT-IR, Thermo Fisher Nicolet iS50, Waltham, MA, USA). The X-ray diffraction patterns of the powders were observed by an X-ray diffractometer with a filtered Cu-Ka X-ray source (XRD, Bruker D8 Advance, Bremen, Germany). The Raman spectra of the samples were obtained using a Renishaw 2000 model Raman spectrometer with a laser power of 100 mW and a 20 s exposure time at 514 nm to investigate the presence of defects in the biochar materials. X-ray photoelectron spectroscopy was used to test the electronic binding energies of the samples (XPS, Thermo Escalab 250Xi^+^, USA). A zeta potential instrument was used to characterize the surface charges of the samples (zeta potential, Zetasizer Nano ZS90, Malvern, UK). N_2_ adsorption–desorption isotherms were used to obtain the porosities of the samples at 77 K (N_2_ adsorption–desorption isotherms, Quantachrome Autosorb iQ2, Boynton Beach, USA). The Brunauer–Emmett–Teller (BET) theory was used to calculate the surface area. The non-local density functional theory (NLDFT) and the Barrett–Joyner–Halenda (BJH) model were used to analyze the pore size distribution of the samples. 

## 4. Conclusions

In this study, LL was used as a biomass carbon source to prepare magnetic lignocellulose-derived hierarchical porous biochar via carbonization, activation, and chemical co-precipitation methods. The specific surface area and total pore volume were 2565.4 m^2^/g and 1.4643 cm^3^/g, and the surface was rich in carbon and oxygen functional groups. The synthetic dye RhB and the antibiotic TH were selected as organic pollutant models to explore the abilities for the removal of organic pollutants, and the results showed good adsorption performances. The maximum adsorption capacities were 1701.7 mg/g for RhB and 1755.9 mg/g for TH, which were higher than most carbon-based adsorbents. After 10 cycles of use, the removal efficiencies were still maintained at more than 70%, showing good stability. Finally, the probable mechanisms affecting the adsorption process were studied, which may include pore filling, π–π interactions, hydrogen bond interactions, and electrostatic attraction. This work not only verified the feasibility of LL as a carbon source for lignocellulose but also prepared a magnetic hierarchical porous biochar with good performances that can better treat RhB and TH, which provided a new idea and direction for the efficient removal of organic pollutants in water.

## Figures and Tables

**Table 1:** The data of N_2_ adsorption–desorption for CITG, CMITG, ITGB, and MITGB.

Samples	S_BET_ (m^2^/g)	P_m_ (nm)	V_total_ (cm^3^/g)
CLL	9.0	11.27	0.0253
LLB-Na	2139.2	2.22	1.1886
LLB-K	1500.8	2.85	1.0692
LLB-MB	2446.6	2.78	1.6990
LLB-MB@Fe_3_O_4_	2565.4	2.28	1.4643

S_BET_, P_m_, and V_total_ represent the BET specific surface area, the mean pore size, and the total pore volume.

**Table 2:** PFK and PSK model parameters for RhB and TH at 303 K.

Adsorbates	Adsorbents	Models	Parameters	*C*_0_ (mg/L)		
				100	200	300
RhB	LLB-MB	PFK	*Q_e_* (mg/g)	941.6	1386.6	1606.5
			*k*_1_ (min^−1^)	0.0087	0.0046	0.0038
			*Q_e.cat_* (mg/g)	904.1	1356.3	1571.5
			*R* ^2^	0.9842	0.9957	0.9928
		PSK	*k*_2_ (g mg^−1^ min^−1^)	0.0006	0.0007	0.0005
			*Q_e.cat_* (mg/g)	943.3	1395.7	1625.2
			*R* ^2^	0.9987	0.9998	0.9995
	LLB-MB@Fe_3_O_4_	PFK	*Q_e_* (mg/g)	998.1	1502.2	1698.2
			*k*_1_ (min^−1^)	0.0033	0.0059	0.0045
			*Q_e.cat_* (mg/g)	988.3	1415.9	1670.0
			*R* ^2^	0.9981	0.9457	0.9965
		PSK	*k*_2_ (g mg^−1^ min^−1^)	0.0006	0.0005	0.0006
			*Q_e.cat_* (mg/g)	1024.2	1513.0	1716.2
			*R* ^2^	0.9964	0.9994	0.9998
TH	LLB-MB	PFK	*Q_e_* (mg/g)	831.4	1298.0	1602.4
			*k*_1_ (min^−1^)	0.0059	0.0066	0.0078
			*Q_e.cat_* (mg/g)	798.3	1237.1	1570.1
			*R* ^2^	0.9845	0.9450	0.9936
		PSK	*k*_2_ (g mg^−1^ min^−1^)	0.0005	0.0005	0.0004
			*Q_e.cat_* (mg/g)	842.0	1321.4	1628.6
			*R* ^2^	0.9998	0.9989	0.9996
	LLB-MB@Fe_3_O_4_	PFK	*Q_e_* (mg/g)	896.1	1453.5	1749.9
			*k*_1_ (min^−1^)	0.0061	0.0035	0.0085
			*Q_e.cat_* (mg/g)	874.8	1413.7	1680.4
			*R* ^2^	0.9939	0.9933	0.9863
		PSK	*k*_2_ (g mg^−1^ min^−1^)	0.0005	0.0004	0.0003
			*Q_e.cat_* (mg/g)	917.5	1469.3	1756.1
			*R* ^2^	0.9983	0.9994	0.9994

**Table 3:** IPD model parameters for RhB and TH at 303 K.

Adsorbates	Adsorbents	Parameters	*C*_0_ (mg/L)		
			100	200	300
RhB	LLB-MB	*k*_3 (5−15 min)_	12.01	16.92	20.92
		*R* ^2^	0.9783	0.9678	0.9950
		*k*_3 (20−60 min)_	1.58	1.13	1.54
		*R* ^2^	0.9865	0.9734	0.8933
	LLB-MB@Fe_3_O_4_	*k*_3 (5−15 min)_	19.43	22.55	20.15
		*R* ^2^	0.9646	0.9529	0.9712
		*k*_3 (20−60 min)_	0.77	1.88	1.19
		*R* ^2^	0.7960	0.9586	0.9067
TH	LLB-MB	*k*_3 (5−15 min)_	14.90	22.13	24.71
		*R* ^2^	0.9686	0.9399	0.9875
		*k*_3 (20−60 min)_	1.70	1.53	1.72
		*R* ^2^	0.9313	0.9160	0.9258
	LLB-MB@Fe_3_O_4_	*k*_3 (5−15 min)_	18.91	22.86	26.14
		*R* ^2^	0.9467	0.9378	0.9856
		*k*_3 (20−60 min)_	1.40	1.78	2.64
		*R* ^2^	0.8833	0.9247	0.9656

**Table 4:** Adsorption isotherm parameters for RhB and TH at 303 K.

Adsorbates	Adsorbents	Types	Parameters	Values
RhB	LLB-MB	Langmuir	*Q_m_* (mg/g)	1531.6
			*K_L_* (L/mg)	0.2699
			*R* ^2^	0.8738
		Freundlich	*K_F_* (mg g^−1^(L mg^−1^)^1/n^)	725.93
			*n_F_*	6.30
			*R* ^2^	0.9972
	LLB-MB@Fe_3_O_4_	Langmuir	*Q_m_* (mg/g)	1582.6
			*K_L_* (L/mg)	1.4446
			*R* ^2^	0.8637
		Freundlich	*K_F_* (mg g^−1^(L mg^−1^)^1/n^)	995.64
			*n_F_*	9.13
			*R* ^2^	0.9957
TH	LLB-MB	Langmuir	*Q_m_* (mg/g)	1788.0
			*K_L_* (L/mg)	0.0456
			*R* ^2^	0.9662
		Freundlich	*K_F_* (mg g^−1^(L mg^−1^)^1/n^)	355.54
			*n_F_*	3.27
			*R* ^2^	0.9991
	LLB-MB@Fe_3_O_4_	Langmuir	*Q_m_* (mg/g)	1844.6
			*K_L_* (L/mg)	0.0870
			*R* ^2^	0.9728
		Freundlich	*K_F_* (mg g^−1^(L mg^−1^)^1/n^)	533.27
			*n_F_*	4.01
			*R* ^2^	0.9912

**Table 5:** Adsorption thermodynamic parameters for RhB and TH.

Adsorbates	Adsorbents	T (K)	∆*G* (kJ/mol)	∆*H* (kJ/mol)	∆*S* (J mol^−1^ K^−1^)
RhB	LLB-MB	293	−6.92	16.23	79.00
		298	−7.34		
		303	−7.86		
		308	−8.21		
		313	−8.50		
	LLB-MB@Fe_3_O_4_	293	−7.70	26.98	118.36
		298	−8.22		
		303	−8.69		
		308	−9.40		
		313	−10.07		
TH	LLB-MB	293	−6.78	6.47	45.22
		298	−7.09		
		303	−7.36		
		308	−7.52		
		313	−7.68		
	LLB-MB@Fe_3_O_4_	293	−7.46	15.45	78.18
		298	−7.86		
		303	−8.31		
		308	−8.73		
		313	−9.02		

**Table 6:** Comparison of biochars with other carbon-based adsorbents.

Adsorbents	*Q_e_* for RhB (mg/g)	*Q_e_* for TH (mg/g)	References
LLB-MB@Fe_3_O_4_	1701.7	1755.9	This work
LLB-MB	1608.1	1602.2	This work
Magnetic K_2_CO_3_-activated carbon	229.9	-	[52]
Magnetic ordered mesoporous carbon	468.0	-	[53]
Sibipiruna activated carbon	630.9	-	[54]
Quinoa-husk-based porous carbon	759.4	-	[40]
Boron-doped activated carbon	1337.2	-	[55]
Zoysia sinica Hance-based carbon	1375.8	-	[56]
Edible fungus substrate porous carbon	1497.0	-	[27]
Corn straw porous carbon	1578.0	-	[26]
Fungal hyphae porous carbon	1912.0	-	[41]
Cow-dung-based biochar	1241.0	1105.0	[28]
Hyphae/starch porous carbon composites	1185.7	1386.0	[51]
*Trichoderma reesei*-based magnetic biochar	-	171.3	[39]
Xanthate-modified activated carbon	-	210.9	[57]
Carbon nanotubes	-	756.2	[58]
Carbon-Fe_3_C/lignin composites	-	760.4	[59]
Magnetic carbon	-	769.4	[60]
Lignin-based biochar	-	1163.0	[61]

## Data Availability

Not applicable.

## Supplementary Materials

The supporting information can be downloaded at: https://www.mdpi.com/article/10.3390/ijms232415703/s1.
