# Supplementary material for: Magnetic Luffa-Leaf-Derived Hierarchical Porous Biochar for Efficient Removal of Rhodamine B and Tetracycline Hydrochloride
Source: Int J Mol Sci. 2022 Dec 11;23(24):15703. doi: 10.3390/ijms232415703 (PMC9779706; doi:10.3390/ijms232415703)
Supplement: Supplementary file 1 [file ijms-23-15703-s001.zip › ijms-2052030-supplementary.pdf]

# Magnetic Luffa-Leaf-Derived Hierarchical Porous Biochar for Efficient Removal of Rhodamine B and Tetracycline Hydrochloride

**Yingjie Su [1,2], Yangyang Zheng [1,2], Meiqin Feng [1,2] and Siji Chen [1,2,*]**

1 College of Life Sciences, Jilin Agricultural University, Changchun 130118, China
2 Key Laboratory of Straw Comprehensive Utilization and Black Soil Conservation, Ministry of Education, Jilin Agricultural University, Changchun 130118, China
* Correspondence: sijichen@jlau.edu.cn

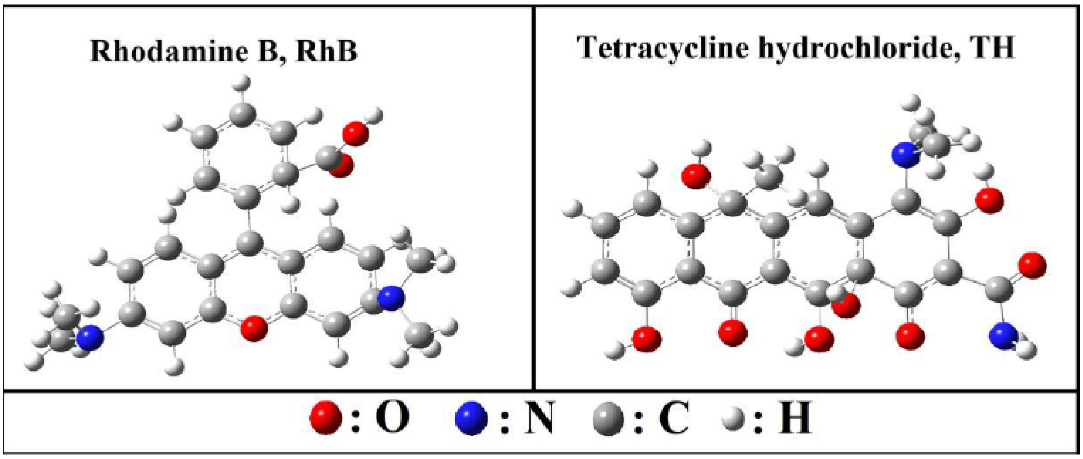


**Figure S1.** The structural formulas of RhB and TH.
